# Bronchopleural Fistula Management With an Amplatzer Duct Occluder: A Comprehensive Case Report and Literature Review

**DOI:** 10.7759/cureus.49431

**Published:** 2023-11-26

**Authors:** Vasileios Leivaditis, Hermann Braun-Lambur, Volker Windmüller, Athanasios Papatriantafyllou, Carmen Huwe, David Lang, Konstantinos Grapatsas, Efstratios N Koletsis, Francesk Mulita, Manfred Dahm

**Affiliations:** 1 Department of Cardiothoracic and Vascular Surgery, Westpfalz-Klinikum, Kaiserslautern, DEU; 2 Department of Pneumonology, Westpfalz-Klinikum, Kaiserslautern, DEU; 3 Department of Cardiology, Westpfalz-Klinikum, Kaiserslautern, DEU; 4 Department of Thoracic Surgery and Thoracic Endoscopy, University Medicine Essen – Ruhrlandklinik, Essen, DEU; 5 Department of Cardiothoracic Surgery, General University Hospital of Patras, Patras, GRC; 6 Department of Surgery, General University Hospital of Patras, Patras, GRC

**Keywords:** endoscopic treatment, amplatzer occluder, postpneumonectomy empyema, bronchopleural fistula, bronchial stump insufficiency

## Abstract

Bronchial stump insufficiency (BSI), also reported as bronchopleural fistula, following pneumonectomy is a rare but potentially devastating complication that can result in substantial morbidity and mortality. Despite advances in thoracic surgical techniques and perioperative care, bronchial stump dehiscence remains a challenging clinical scenario, especially when associated with severe infections and compromised patient conditions. Traditional surgical re-intervention to address this complication may carry significant risks and might be contraindicated in certain patients. As a result, innovative interventions are necessary to address these challenging cases effectively. In this report, we present an interventional endoscopic technique using an Amplatzer Duct occluder for the successful management of BSI in a 55-year-old male patient with a complex medical history.

## Introduction

Pneumonectomy is a major thoracic surgical procedure often performed for various conditions, including malignancies, extensive infections, and end-stage lung diseases. Despite improvements in surgical techniques and perioperative care, complications can still arise, and bronchial stump insufficiency (BSI) remains a rare yet serious occurrence [1]. BSI is characterized by the breakdown of the bronchial suture line, leading to the development of bronchopleural fistulas (BPFs) and the subsequent communication between the bronchial tree and the pleural cavity [2].

The primary etiology of BPF predominantly stems from post-pneumonectomy procedures, with incidence rates estimated to range from 4.5% to 20% following total pneumonectomy and from 0.5% to 1% after lobectomy [1]. However, it is important to note that the mortality rate associated with BPF after pneumonectomy can be alarmingly high, ranging from 18% to 50% [2]. Patients who underwent right pneumonectomy and right lower lobectomy demonstrated an elevated incidence of BPF [3]. Additional causative factors encompass necrotizing infections such as pulmonary tuberculosis, pneumonia, empyema, the application of chemotherapy or radiation therapy, and thoracic trauma [4].

After a pneumonectomy, BPF can be classified into three distinct stages based on the modified Le Brigand classification, according to the timing of onset. Early onset manifests within one to seven days after the surgical procedure, intermediate onset occurs between eight to 30 days post-surgery, and late onset arises more than 30 days after the surgical intervention [5].

The clinical consequences of BSI are substantial, as it can lead to persistent air leaks, pleural empyema, postoperative infections, and even fatal septic complications [1,6]. Traditional management of BSI typically involves reoperation with surgical revision, closure of the defect, and tissue reinforcement such as muscle flaps, or omentum transposition [6]. The reported success rate of surgery for BPF closure has been more than 85% [6]. However, in some cases, reoperation may not be feasible due to the patient's medical condition, underlying comorbidities, or the extent of the primary disease.

In recent years, interventional endoscopic techniques have emerged as potential alternatives for managing various thoracic conditions [1]. In particular, the off-label use of the Amplatzer occluders, originally designed for other uses, such as transcatheter closure of atrial septal defects (ASDs), has shown promise in treating BPFs [7]. The occluder is a self-expanding device made of nitinol wire mesh and polyester fabric that can be deployed endoscopically to occlude abnormal communications.

Given the scarcity of reported cases using Amplatzer occluders for BSI, there is a lack of consensus on its efficacy and safety. Therefore, we present this unique case report to contribute to the existing body of knowledge and highlight the successful application of this novel salvage interventional endoscopic technique. To the best of our knowledge, this is the first reported case of using this particular technique with this device in such a scenario.

## Case presentation

This report presents the case of a 55-year-old male patient with a cachectic general condition and a medical history of amphetamine and heroin abuse, hepatitis C, and multiple right frontal cerebral infarctions. The patient was admitted to a local hospital with an acute abdomen, and a subsequent median laparotomy revealed evidence of perforation of the sigmoid colon, necessitating immediate intervention in septic shock. During the procedure, a four-quadrant peritonitis was identified, and a colostomy was created. Secondary closure of the abdominal wall was performed two weeks later. However, persistent infection prompted a computed tomography (CT) scan, which revealed a pronounced pneumonia in the left lung with abscess formation in the left lower lobe, indicative of a destroyed lobe. The patient was transferred to our clinic for further thoracic surgical treatment.

Surgical treatment

Intraoperative findings showed a completely destroyed lower lobe and a severely pneumonic infiltrated upper lobe, accompanied by a pronounced stage III pleural empyema. An open lobectomy of the left lower lobe was performed, and the pleural empyema was effectively addressed. Cultures from intraoperative samples and tracheal secretions revealed highly resistant *Pseudomonas aeruginosa*, necessitating tailored antibiotic therapy based on the antibiogram.

Postoperative course

**Figure 1:**
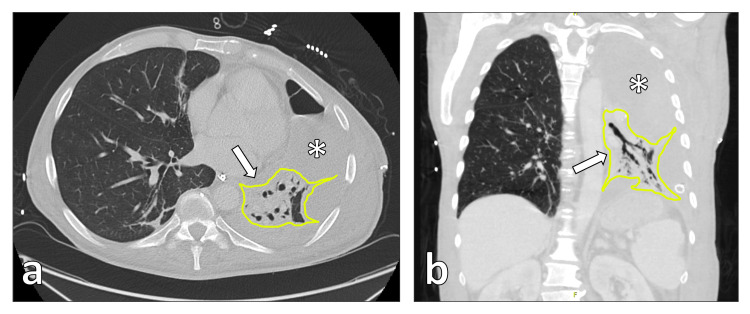
CT scan showing postoperative empyema (asterisk) and abscess formation in the remaining left upper lobe (arrow). (a) Transversal view. (b) Coronary reconstruction. CT, computed tomography

Despite aggressive antibiotic management, daily bronchial toilet, and diligent patient positioning, the upper lobe's recovery remained elusive, with subsequent CT scans indicating abscess formation (Figure 1). As shown in Figure 1, the remaining upper lobe is atelectatic without vital parenchyma, indicating the initial stages of abscess formation. There is also concomitant pleural effusion, which can be correlated with pleural empyema. Consequently, a rethoracotomy involving a lobectomy of the left upper lobe, akin to a residual pneumonectomy, was performed on the 16th postoperative day. Unfortunately, the cachectic patient's condition precluded the availability of suitable intraoperative materials (e.g., mediastinal fatty tissue, intercostal muscles) to adequately cover the bronchus stump. Furthermore, mobilizing a diaphragmatic flap was avoided to prevent intraperitoneal spread of the infection. During the rethoracotomy, an infected hematoma was also encountered, necessitating complete clearance, followed by pleural cavity lavage with antiseptic solutions.

**Figure 2:**
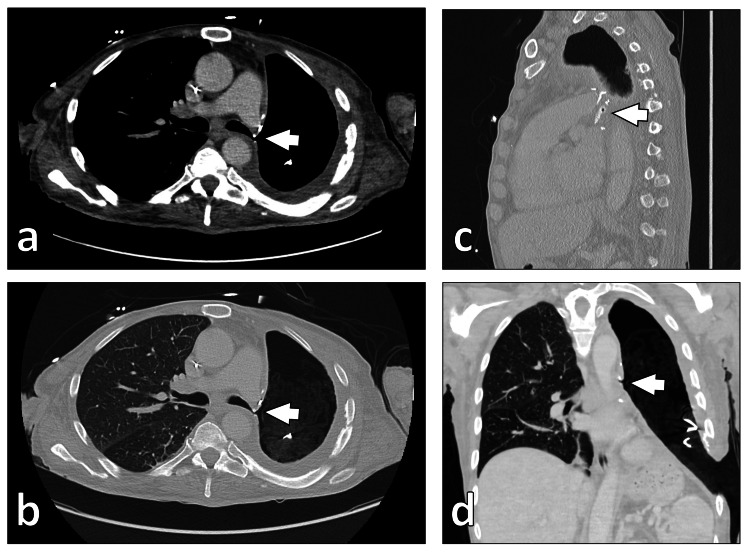
CT scan demonstrating bronchial stump insufficiency (indicated by the arrow) following pneumonectomy. (a, b) Transversal views. (c) Sagittal reconstruction. (d) Coronary reconstruction. CT, computed tomography

Subsequently, despite aggressive antibiotic therapy, highly resistant pseudomonads persisted in both the pleural fluid and tracheal secretion, and a postoperative post-pneumonectomy empyema gradually developed. The clinical indication was made to proceed with pleural empyema removal and terminal thoracostomy creation, which was performed on the 21st postoperative day through partial resection of the seventh rib. Vacuum-assisted closure was primarily employed for treating the pleural cavity and later with daily dressing changes using sterile surgical towels. Throughout the weaning process from the respirator, a bronchoscopic and CT scan evaluation revealed bronchus insufficiency (Figure 2). Due to the inherent high-risk nature of further reoperation, both the patient and legal guardian declined additional surgical intervention.

Interventional treatment

**Figure 3:**
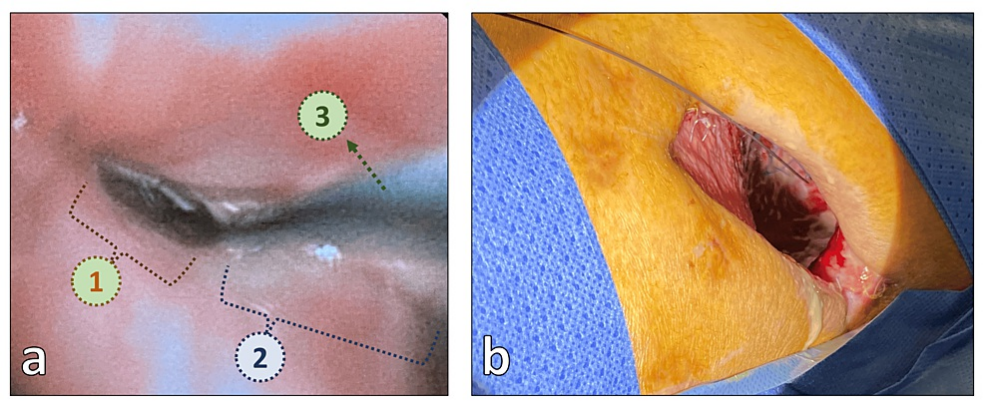
(a) Bronchoscopic view of the stump insufficiency: 1. The insufficient section of the stapler line. 2. The fully healed part of the stapler line. 3. The guide wire passed through the bronchopleural fistula, entering the pleural cavity. (b) The guide wire, which was passed through the hole of the bronchial stump, was retrieved from the pleural cavity through the thoracostomy hole.

**Figure 4:**
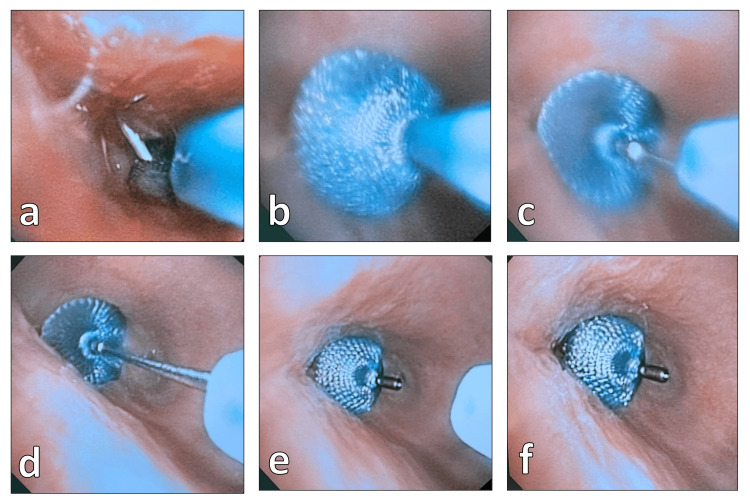
The procedure for implanting the Amplatzer duct occluder through the bronchopleural fistula. (a) Insertion of the device through the defect hole over the guide wire. (b) Initiation of the deployment of the device. (c) Completion of the deployment in the defect area. (d) Withdrawal of the application system. (e) Release of the implant from the deployment device. (f) Final evaluation demonstrates optimal positioning of the occluder with no remaining insufficiency.

**Figure 5:**
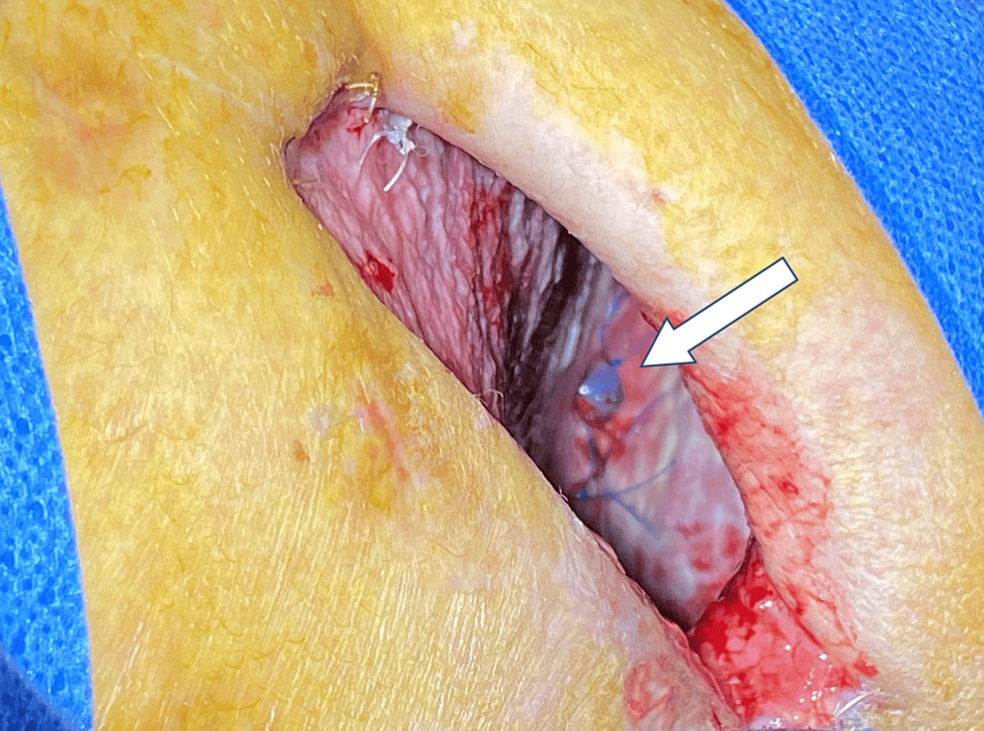
View of the distal part of the Amplatzer duct occluder (indicated by the arrow) in the pleural cavity through the thoracostomy hole.

Following interdisciplinary discussions, an off-label interventional treatment approach involving endoscopic implantation of a 4/6 mm Amplatzer® Duct Occluder II (Abbott Medical GmbH, Wetzlar, Germany) closure system was decided upon. On the 78th postoperative day, the procedure was carried out, involving the insertion of a guide wire via the bronchoscope's working channel through the hole in the bronchus stump into the pleural cavity and then out through the thoracostoma (Figure 3). The occluder was successfully implanted over the guide wire, leading to gradual improvements in the patient's condition, with observed reductions in leakage (Figures 4, 5).

Outcome

**Figure 6:**
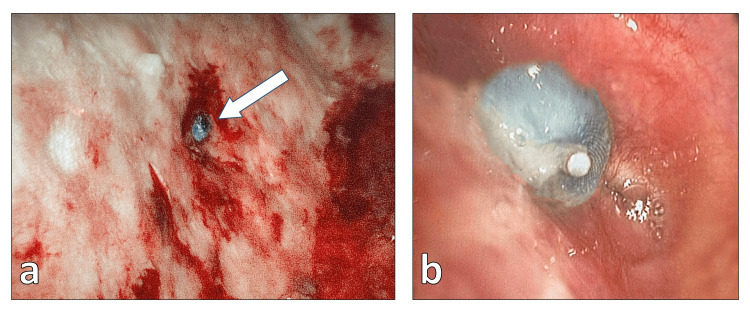
(a) Thoracoscopic view of the distal part of the Amplatzer duct occluder (indicated by the arrow) in the pleural cavity, showing well-developed granulation tissue around the occluder with stable integration of the implant. (b) Bronchoscopic view of the proximal part of the Amplatzer occluder in the bronchial stump. The implant material is also securely integrated, with no signs of further insufficiency.

After successful weaning from the ventilator, the patient was decannulated on the 83rd postoperative day, following which he was transferred to a standard ward on the 92nd postoperative day. Subsequent physiotherapy and respiratory exercises were implemented, leading to positive outcomes during bronchoscopic and thoracoscopic assessments five weeks post-occluder implantation, demonstrating favorable epithelialization and defect coverage with granulation tissue (Figure 6). The patient's progress allowed for transfer to a supervised apartment on the 118th postoperative day.

## Discussion

BSI is a life-threatening complication, and its management requires a personalized approach [1]. In this case, the decision to use an Amplatzer occluder as a salvage interventional endoscopic technique was based on the patient's preference to avoid further surgical interventions. The successful outcome and the absence of similar reported cases in the literature highlight the novelty and potential usefulness of this off-label approach.

Treatment options for BPFs range from conservative measures to extensive surgical revisions [1,6,7]. Conservative approaches involving thoracic drainage do not directly cure BPF, but they can aid in partial closure of small fistulas (>3 mm in diameter) by removing pus and thus maintaining fluid balance [8].

Bronchoscopic interventions have gained prominence as reliable alternatives in cases where surgical interventions are unsuitable due to their adaptability and versatility [1,7]. These interventions encompass various techniques, such as bronchoscopic placement of blocking agents, ASD/ventricular septal defect (VSD) occluders, airway stents, endobronchial valves, vascular occlusion coils, adhesive tissue, fibrin glue, and endobronchial Watanabe spigots [1,6,7]. Additionally, advancements in mesenchymal stem cell transplantation technology and three-dimensional printed stents have contributed to BPF treatment, although more research is required to assess their long-term benefits [1].

The use of ASD/VSD occluders, initially designed for heart-related conditions, has shown promise in treating postpneumonectomy BPF. Early instances include Zhang et al.'s use of a self-expandable double umbrella-shaped occluder in 2007 [9] and Kramer et al.'s effective treatment of BPF using Amplatzer ASD devices [10]. Subsequent isolated cases have also reported positive outcomes using ASD occluders [7,11,12]. In 2012, Krumpolcova et al. reported the closure of a BPF using a patent foramen ovale (PFO) occluder [13]. The same year, Fruchter et al. successfully employed an Amplatzer vascular plug originally designed for vascular structure closure [14].

Fruchter et al.'s study involving 31 BPF patients revealed that 96% experienced immediate symptomatic relief with Amplatzer occluders, and they remained stable during follow-up, extending up to 18 months [15]. In 2016, Lin et al. documented a case of BPF treatment with a dumbbell-shaped closure device [16]. Although waist diameter selection for occluders lacks a standardized guideline, Motus et al. recommended a waist 30% wider than the fistula diameter, based on a retrospective study with 13 patients, affirming the reliability of ASD occluder use for bronchial fistula treatment [8]. Zhang et al. also introduced a “sheath-free method” for ASD occluder placement, enhancing clinical convenience and efficiency [17]. Bai et al. reported on six BPF patients who underwent bronchoscopic closure using VSD occluders, demonstrating its safety and effectiveness. They emphasized stabilizing the BPF by treating underlying diseases and providing nutritional support to those undergoing VSD occluder closure [6].

Treating BPF often necessitates a multidisciplinary approach, involving both thoracic surgery and respiratory intervention [1]. While no uniform guidelines recommend standardized BPF treatment, bronchoscopic methods are typically recommended for fistulas ≤8 mm in diameter, with the closure of larger fistulas remaining a challenging endeavor [1,6]. It is essential to recognize that bronchoscopic treatment plays a crucial role in managing BPF, and treatment approaches should be tailored to each patient's unique condition [7].

The future perspectives of bronchoscopic techniques for treating BPFs, particularly with the use of devices like Amplatzer occluders, appear promising. As these techniques continue to evolve, they hold the potential to offer less invasive and more patient-friendly alternatives to traditional surgical interventions. Further research and clinical studies are needed to establish standardized protocols, refine occluder selection criteria, and enhance long-term monitoring of patients. With ongoing advancements in device technology, improved procedural efficiency, and an expanded understanding of patient selection, bronchoscopic treatments using devices like Amplatzer occluders are likely to play an increasingly important role in the comprehensive management of BPFs, providing patients with effective, minimally invasive solutions.

## Conclusions

This case demonstrates the successful application of an Amplatzer occluder via an endoscopic approach as a salvage technique for managing BSI after pneumonectomy in a patient with complex medical comorbidities. It emphasizes the importance of considering alternative strategies when managing challenging cases of BSI, especially in patients with complex medical histories and limited surgical options. The successful application of the Amplatzer PFO occluder warrants further investigation and provides valuable insights into potential non-surgical interventions for selected patients. Nevertheless, caution should be exercised when using off-label interventions, and additional reported cases and prospective studies are essential to validate the safety and efficacy of this approach.
